# Effect of tacrolimus with mycophenolate mofetil or cyclophosphamide on the renal response in systemic lupus erythematosus patients

**DOI:** 10.1186/s41927-024-00439-x

**Published:** 2024-12-18

**Authors:** Siqin Sun, Xueyi Zhang, Qingqing Guo, Xiaojun Tang, Wei Shen, Jun Liang, Genhong Yao, Linyu Geng, Shuai Ding, Hongwei Chen, Hong Wang, Bingzhu Hua, Huayong Zhang, Xuebing Feng, Ziyi Jin, Lingyun Sun

**Affiliations:** 1 Department of Rheumatology and Immunology, Nanjing Drum Tower Hospital, Basic Medicine and Clinical Pharmacy School, China Pharmaceutical University, Nanjing, China; 2 Department of Rheumatology and Immunology, Nanjing Drum Tower Hospital, Affiliated Hospital of Medical School, Nanjing University, 321 Zhongshan Road, Nanjing, 210008 China; 3 Rheumatology Medical Center and Stem Cell Intervention Center, Department of Rheumatology and Immunology, Nanjing Drum Tower Hospital, Affiliated Hospital of Medical School, Nanjing University, Nanjing, 210008 P.R. China; 4 Department of Rheumatology and Immunology, China Pharmaceutical University Nanjing Drum Tower Hospital, 321 Zhongshan Road, Nanjing, 210008 China

**Keywords:** Lupus nephritis, Tacrolimus, Mycophenolate mofetil, Cyclophosphamide, Dose-response

## Abstract

**Objective:**

This study aimed to determine the therapeutic efficacy of tacrolimus (TAC) with mycophenolate mofetil (MMF) or cyclophosphamide (CYC) on the renal response in systemic lupus erythematosus (SLE) patients.

**Methods:**

A retrospective cohort study based on medical data was conducted among SLE patients who took at least one of the following medicines in 2010–2021: TAC, MMF and CYC. The odds ratio (OR) and 95% confidence interval (CI) were calculated, and the synergistic interaction was estimated using logistic regression models.

**Results:**

Among 793 SLE patients, 27.9% patients (221 cases) achieved CR after at least 3 months. The TAC use was positively associated with CR with an adjusted OR (95% CI) of 2.82 (1.89, 4.22) overall and in subgroups of SLE patients with SLEDAI scores > 12, moderate or severe urinary protein and comorbidities. The dose-response effect on CR was also observed at TAC doses greater than 4 mg/d and more than 180 days, with adjusted ORs (95% CIs) of 5.65 (2.35, 13.55) and 3.60 (2.02, 6.41), respectively. Moreover, the combined effect of TAC with MMF or CYC was better than that of monotherapy, there was significant synergistic interactions with adjusted ORs (95% CIs) of 2.43 (1.20, 4.92) and 3.14 (1.49, 6.64), respectively, and similar results were observed for the combination of different doses of TAC with MMF or CYC.

**Conclusion:**

TAC can effectively alleviate the condition of patients with SLE and may interact with MMF or CYC, which suggests that the combination therapy of TAC with MMF or CYC may produce greater benefits for patients with SLE.

**Trial registration:**

This is a purely observational study that does not require registration.

**Supplementary Information:**

The online version contains supplementary material available at 10.1186/s41927-024-00439-x.

## Introduction

Systemic Lupus Erythematosus (SLE) is a complex autoimmune disease that affects multiple systems, organs, and tissues [1]. Renal involvement is a serious complication of SLE. Approximately 40–60% of patients with SLE will exhibit lupus nephritis (LN), and approximately 26% of patients with LN will progress to end-stage renal disease (ESRD) [2, 3]. The main clinical manifestations of renal involvement include urinary protein, haematuria, cellular casts, decreased glomerular filtration function, and elevated serum creatinine levels. The Chinese guidelines recommend assessing the disease activity at least once a month for patients with active SLE and once every 3–6 months for patients with stable SLE [4]. The primary treatment options for SLE include glucocorticoids (GCs), immunosuppressants, and biological agents [5]. Tacrolimus (TAC), which is a calcineurin inhibitor, works by binding to the FK506 binding protein 12 in T-lymphocytes and consequently inhibiting the T-cell activation and calcineurin [6]. It also suppresses B-cell, plasma cell, and CD40 receptor signalling [7–10]. TAC is effective in improving the renal function in SLE patients [7, 8]. 

Moreover, multitarget therapy has demonstrated significant efficacy in LN patients, and TAC is often used in combination with mycophenolate mofetil (MMF) and cyclophosphamide (CYC) [9–11]. The combination of TAC with other drugs can reduce the dosages of TAC and other drugs, drug-related organ damage and infections [12–14]. Multiple clinical studies have shown that a treatment regimen that combines TAC with MMF or CYC has significant renal therapeutic effects on LN patients who fail to respond to monotherapy [15, 16]. 

However, the dosage of TAC in combination with other drugs remains controversial, and few studies have explored the duration of TAC use [12, 13]. Although previous studies involved drug combinations, they rarely assessed the impact of drug synergy on the renal response. In addition, previous studies involved fewer than 400 patients, and the synergistic effect of TAC with MMF or CYC was rarely estimated in Chinese SLE patients [17]. Thus, we performed a retrospective cohort study to investigate the clinical efficacy of TAC and its synergy with MMF or CYC in Chinese patients with SLE.

## Methods

### Study design and participants

**Fig. 1:**
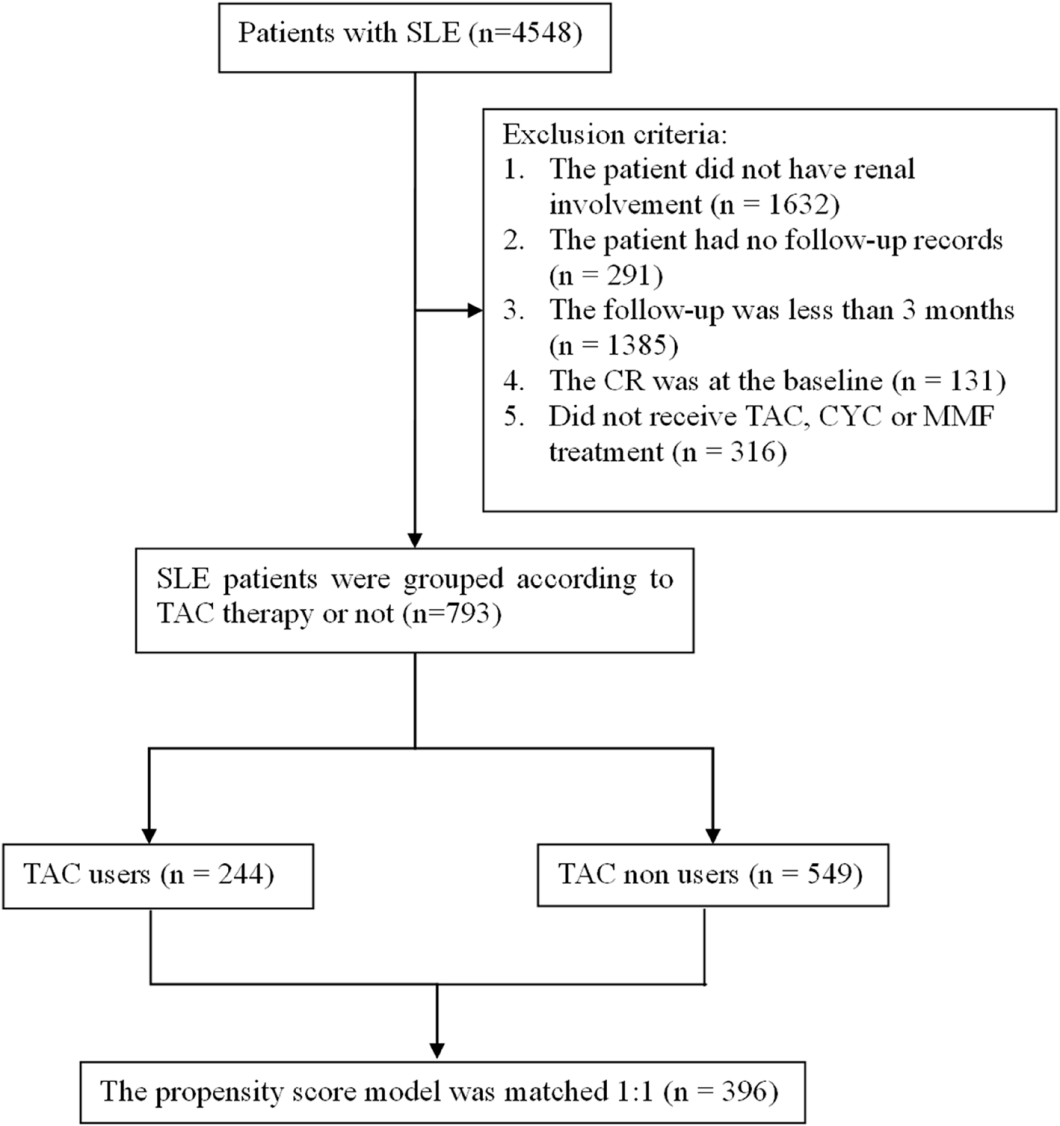
Selection of patients for the study

This retrospective cohort study was conducted at the Affiliated Drum Tower Hospital, Nanjing University Medical School. Figure 1 shows the process of enrolling participants. We gathered the medical records of SLE patients who visited the rheumatology and immunology departments between January 1, 2010 and December 31, 2021. To be included in the study, patients had to meet at least four of the classification criteria for SLE, as revised and updated by the American College of Rheumatology [14]. In accordance with the LN guidelines, we determined the following criteria for renal involvement in SLE patients: (1) 24-hour protein measurement > 500 mg or a urine protein/creatinine ratio > 30 mg/mmol; (2) The patient had cellular casts and a pathological tube pattern; (3) The patient had active urinary sediment [18]. The exclusion criteria were: the patient did not have renal involvement, medical information was incomplete, there was no follow-up record or the follow-up was less than 3 months, the CR was at the baseline, and the patient was not treated with TAC, CYC or MMF.

### Data collection and definition

We gathered data from the medical records of both inpatients and outpatients with SLE, including demographics, diagnostic information, physical exams, SLEDAI scores, laboratory test data, medication records, pathology reports, and imaging information. The missing laboratory test data and medication information of hospitalized patients were supplemented by the latest outpatient medical information within three months before and after hospitalization. The comorbidities of the patients included hypertension, hyperlipidaemia, diabetes, secondary Sjogren’s syndrome and cancer. We established the time of the patient’s hospitalization as the baseline and defined the endpoint as the occurrence of renal response or the final visit after at least 3 months of continuous follow-up. TAC users were defined as patients who had initiated TAC usage since the baseline, whereas TAC non-users were those who did not use TAC from the baseline through the endpoint of the study. In the therapeutic regimen that involved a combination of TAC and other immunosuppressants, CYC and MMF may be sequentially administered over the course of the observation period instead of being simultaneously given. The use of TAC in this study was attributed to 32.7% (80) new cases of renal damage or disease onset, 14.8% (36) relapse, and 52.5% (128) DMARD failure. Several diseases that occurred during or before this period but had not been cured were defined as comorbidities. The patients used renal-protective agents, including beraprost sodium tablets, haikunshenxi capsules, huangkui capsules, niaoduqing granules, shenfukang, shenyankangfu, lanthanum carbonate, sevelamer carbonate and pioglitazone hydrochloride and glimepiride. The urinary protein levels were divided into three groups: mild urinary protein: 24-hour urinary protein less than 1000 mg; moderate urinary protein: 24-hour urinary protein of 1000–3500 mg; severe urinary protein: 24-hour urinary protein greater than 3500 mg [19]. The patients were treated with different types of GCs, and we converted all GC doses to prednisone doses (5 mg prednisone = 4 mg methylprednisolone = 0.75 mg dexamethasone = 20 mg hydrocortisone). The CR was defined as follows: (a) normal urinary protein (24-hour protein measurement excretion less than 500 mg/day or urinary protein negative or weakly positive); (b) serum creatinine was normal or increased by no more than 25% of the baseline.

### Statistical analysis

The data were analysed using the IBM SPSS STATISTICS 25.0 software. Shapiro-Wilk test was applied to test the normality of continuous variables. Abnormally distributed variables are described by the median and interquartile range (IQR) and were compared using Mann-Whitney U test. Categorical variables are presented as counts (percentages) and were compared via the χ [2] test or Fisher’s exact test. A binary unconditional logistic regression model was used to calculate the odds ratio (OR) and corresponding 95% confidence interval (CI) between treatment with TAC and overall CR. The OR and *P*-value for trend were estimated for each exposure category and dose of TAC using dummy variables and ordinal coding. Stratified analyses were conducted based on factors such as sex, age, SLEDAI score at the baseline, disease course, comorbidities, urinary protein, laboratory tests, and combined drug use.

We used a binary logistic regression model to assess the potential synergistic interaction between TAC and MMF or CYC. The confounding factors in the binary regression analysis were sex, age, SLE disease period, comorbidities, SLEDAI score, renal-protective agents, HCQ treatment, GC dose, abnormal urinary protein, abnormal serum creatinine and other immunosuppressants. These adjusted variables were excluded when they were used as stratified variables. To eliminate the influence of confounding factors and verify the credibility of the results, we used a binary logistic regression analysis for propensity score matching and controlled for sensitivity factors that significantly impacted the outcome. The propensity score model included the recipient’s age, sex, SLEDAI score, abnormal urinary protein, and abnormal creatinine rate. Then, we conducted a secondary analysis of the main results associated with the relationship between TAC and SLE patients.

## Results

### Characteristics of the study patients

Among 793 SLE patients, 244 patients were in the TAC use group, and 549 patients were in the TAC non-use group. Table 1 shows the demographic and baseline clinical characteristics of the patients. Compared with TAC nonusers, TAC users tended to be younger and had greater body weights. The median age (IQR) for TAC non-users and users was 37.0 (28.0, 50.0) and 27.0 (22.0, 35.0) (*P* < 0.001), respectively. The median weight (IQR) was 55.00 (49.00, 63.00) for TAC non-users and 57.63 (55.00, 65.50) for TAC users (*P* < 0.001). The dose of CYC or MMF with TAC was lower than that used alone. Compared with TAC non-users, TAC users had significantly higher median course of disease period, SLEDAI score, and severe urinary protein proportions but lower proportions of moderate urinary, abnormal serum creatinine, anti-dsDNA, and C4. TAC non-users were more likely to use renal-protective agents, GCs, HCQ, CYC and other immunosuppressants. MMF was more prevalent in the TAC user group.

**Table 1:** Baseline clinical characteristics of the SLE patients

Variable	TAC nonusers	TAC users	*P*
Total	*n* = 549	*n* = 244	—
Gender (Female), n (%)	487(88.7)	226(92.6)	0.098
Age, M (IQR), years	37.0(28.0,50.0)	27.0(22.0,35.0)	**< 0.001**
Weight, M (IQR), kg	55.0(50.0,62.0)	57.6(55.0,65.0)	**< 0.001**
SLE disease period, M (IQR), years	0.5(0.0,6.0)	1.0(0.0,3.0)	**0.033**
Renal biopsy, n (%)	52(9.5)	37(15.2)	**0.028**
SLEDAI score, M (IQR)	14.0(8.3,20.0)	16.0(12.0,21.0)	**< 0.001**
Follow-up duration, M (IQR), days	126(94.0,221.0)	118.0(91.0,199.0)	0.473
Comorbidities, n (%)			
All	465(84.7)	217(88.9)	0.121
Hyperlipemia	206(37.5)	130(53.3)	**< 0.001**
Hypertension	316(57.6)	167(68.4)	**0.004**
Diabetes	46(8.4)	20(8.2)	1.000
Tumour	30(5.5)	11(4.5)	0.728
Secondary Sjogren’s syndrome	17(3.1)	2(0.8)	0.075
Indicators of renal involvement, n (%)			
Urinary protein			
Mild	129(23.5)	31(12.7)	0.607
Moderate	141(25.7)	55(22.5)	**< 0.001**
Severe	279(50.8)	158(64.8)	**< 0.001**
Pathological tube pattern	126(32.0)	62(25.4)	0.470
Abnormal serum creatinine	263(48.5)	79(32.4)	**< 0.001**
Abnormal eGFR	155(80.3)	154(77.4)	0.537
Anti-dsDNA positive, n (%)	400(72.9)	42(17.2)	**0.003**
Low complement, n (%)			
C3 ≤ 0.8 g/L	373(74.9)	175(71.7)	0.374
C4 ≤ 0.2 g/L	404(81.1)	151(61.9)	**< 0.001**
Treatments			
TAC dose, M (IQR), mg/d	—	3.0(2.0,3.0)	—
MMF, n (%)	208(24.1)	91(37.3)	0.937
MMF dose, M (IQR), g/d	1.0(1.0,1.5)	1.0(0.8,1.5)	**0.001**
CYC, n (%)	419(76.3)	108(44.3)	**< 0.001**
CYC dose, M (IQR), g/m^2^	0.4(0.4,0.4)	0.4(0.2,0.4)	**0.012**
GCs, n (%)	546(99.5)	207(84.8)	**< 0.001**
GCs maintenance dose, M (IQR), mg/d	30.0(20.0,40.0)	30.0(15.0,50.0)	0.814
Renal-protective agents, n (%)	166(30.2)	37(15.2)	**< 0.001**
HCQ, n (%)	488(88.9)	179(73.4)	**< 0.001**
Other immunosuppressants, n (%)	420(76.5)	138(56.6)	**< 0.001**
Leflunomide	116(21.1)	63(25.8)	
Methotrexate	11(2.0)	3(1.2)	
Tripterygium wilfordii	34(6.2)	10(4.1)	
Azathioprine	4(0.7)	8(3.3)	
Metronidazole	0(0.0)	2(0.8)	
Sunitinib	20(3.6)	9(3.7)	

*M (IQR)* Median (interquartile range), *eGFR* estimated glomerular filtration rate, *Anti-dsDNA* Anti-double stranded DNA, *GCs* Glucocorticoids, *HCQ* Hydroxychloroquine, *MMF* Mycophenolate mofetil, *CYC* Cyclophosphamide

### Effect of TAC use on CR

CR was achieved in 27.9% (221) of all SLE patients, and the use of TAC was positively associated with CR with an adjusted OR (95% CI) of 2.82 (1.89, 4.22). A TAC dose ≥ 4 mg/d and TAC use > 180 days were found to be associated with an increased likelihood of CR compared to non-users with adjusted ORs (95% CI) of 5.65 (2.35, 13.55) and 3.60 (2.02, 6.41), respectively. A dose-response relationship was also observed between CR and the dose (*P* for trend < 0.001) and duration (*P* for trend < 0.001) of TAC use (Table 2). After the propensity score matching, the age, SLEDAI score, comorbidities, abnormal urinary protein, and abnormal creatinine rates were balanced between TAC users and non-users. In total, 396 patients were matched, including 198 patients in the TAC use group and 198 patients in the TAC non-use group, and 32.1% achieved CR (Supplementary Table 1). The use of TAC was positively correlated with CR, where the adjusted OR (95% CI) was 2.40 (1.48, 3.88). A dose-response relationship between the TAC dosage and duration of use and CR was also observed (Table 2).

**Table 2:** Complete response rates according to the TAC use in SLE patients and these patients after propensity score matching

Variable	Non-CR, *n* (%)	CR, *n* (%)	OR	Adjusted OR
			(95% CI)	(95% CI)
**All patients**	***n* = 572**	***n* = 221**		
TAC treatment				
No	431(78.5)	118(21.5)	1.00	1.00
Yes	141(57.8)	103(42.2)	2.67(1.93,3.7)	2.82(1.89,4.22)
TAC dose				
No	431(78.5)	118(21.5)	1.00	1.00
≤ 2 mg/d	88(61.1)	56(38.9)	2.32(1.57,3.44)	2.42(1.53,3.84)
3 mg/d	43(57.3)	32(42.7)	2.72(1.65,4.49)	2.92(1.65,5.16)
≥ 4 mg/d	10(40.0)	15(60.0)	5.48(2.40,12.51)	5.65(2.35,13.55)
*P* for trend			< 0.001	< 0.001
TAC length of use				
No	431(78.5)	118(21.5)	1.00	1.00
≤ 90 days	37(61.7)	23(38.3)	2.27(1.30,3.97)	2.48(1.33,4.63)
91–180 days	67(58.8)	47(41.2)	2.56(1.68,3.92)	2.60(1.59,4.23)
> 180 days	37(52.9)	33(47.1)	3.26(1.95,5.43)	3.60(2.02,6.41)
*P* for trend			< 0.001	< 0.001
**PSM**	*n* = 572	*n* = 221		
TAC treatment				
No	155(78.3)	43(21.7)	1.00	1.00
Yes	114(57.6)	84(42.4)	2.66(1.71,4.12)	2.40(1.48,3.88)
TAC dose				
No	155(78.3)	43(21.7)	1.00	1.00
≤ 2 mg/d	74(63.2)	43(36.8)	2.09(1.26,3.47)	1.88(1.09,3.23)
3 mg/d	33(53.2)	29(46.8)	3.17(1.73,5.79)	2.95(1.53,5.67)
≥ 4 mg/d	7(36.8)	12(63.2)	6.18(2.29,16.65)	5.08(1.84,14.04)
*P* for trend			< 0.001	< 0.001
TAC length of use				
No	155(78.3)	43(21.7)	1.00	1.00
≤ 90 days	30(65.2)	16(34.8)	1.92(0.96,3.85)	1.64(0.79,3.38)
91–180 days	52(57.1)	39(42.9)	2.70(1.58,4.62)	2.60(1.47,4.60)
> 180 days	32(52.5)	29(47.5)	3.27(1.78,5.99)	2.88(1.49,5.54)
*P* for trend			< 0.001	< 0.001

The confounding factors in the multivariate regression analysis of all patients were sex (female = 1, male = 0), age (continuous), SLE disease period (continuous), comorbidities (yes = 1, no = 0), SLEDAI score (continuous), abnormal urinary protein (yes = 1, no = 0), abnormal serum creatinine (yes = 1, no = 0), renal-protective agents (yes = 1, no = 0), MMF treatment (yes = 1, no = 0), CYC treatment (yes = 1, no = 0), HCQ treatment (yes = 1, no = 0), GCs dose (continuous) and other immunosuppressants (yes = 1, no = 0)

The confounding factors in the multivariate regression analysis of patients after the propensity score matching were sex (female = 1, male = 0), SLE disease period (continuous), renal-protective agents (yes = 1, no = 0), MMF treatment (yes = 1, no = 0), CYC treatment (yes = 1, no = 0), HCQ treatment (yes = 1, no = 0), GCs dose (continuous) and other immunosuppressants (yes = 1, no = 0)

We also conducted a stratified analysis of 14 factors, such as sex, age, SLE disease period, SLEDAI score, urinary protein, comorbidities, laboratory tests, and therapeutic drugs (Table 3). The CR rate was significantly higher in the TAC use group than in the non-use group across most strata but was not significantly different among male patients, mild urinary protein patients, patients with low complement 3 levels, patients without defined comorbidities, those receiving renal-protective agent treatment and those rejecting HCQ treatment.

**Table 3:** Complete response rates using TAC in subgroups of SLE patients

Variable	Non-users	TAC Users		
	Non-CR/CR	Non-CR/CR	OR (95% CI)	Adjusted OR (95% CI)
Gender				
Male	48/14	14/4	0.98(0.28,3.46)	1.01(0.18,5.53)
Female	383/104	127/99	2.87(2.04,4.04)	3.04(1.99,4.66)
Age, years				
≤ 30	148/41	90/67	2.69(1.68,4.29)	3.08(1.71,5.56)
> 30	283/77	51/36	2.59(1.58,4.26)	2.76(1.52,4.99)
SLE disease period, years				
≤ 2	261/75	101/71	3.08(1.71,5.56)	2.45(1.64,3.64)
> 2	170/43	40/32	2.76(1.52,4.99)	3.16(1.78,5.61)
SLEDAI score				
≤ 12	199/57	54/30	1.94(1.14,3.31)	1.93(1.00,3.79)
> 12	253/61	87/73	3.19(2.10,4.86)	3.42(2.02,5.80)
Urinary protein				
Mild	89/40	20/11	1.22(0.54,2.79)	0.95(0.27,3.32)
Moderate	112/29	26/29	4.31(2.21,8.41)	4.62(1.94,11.00)
Severe	230/49	95/63	3.11(2.00,4.85)	2.80(1.63,4.81)
Complement 3, g/L				
≤ 0.8	296/77	102/73	2.75(1.86,4.07)	2.91(1.81,4.68)
> 0.8	89/36	39/30	1.90(1.03,3.51)	1.99(0.85,4.64)
Complement 4, g/L				
≤ 0.2	312/92	79/72	3.09(2.08,4.59)	2.90(1.80,4.67)
> 0.2	73/21	62/31	1.74(0.91,3.33)	3.26(1.23,8.65)
Comorbidities				
No	60/24	15/12	2.00(0.82,4.89)	1.85(0.38,8.91)
Yes	371/94	126/91	2.85(2.00,4.05)	3.08(2.01,4.73)
Renal-protective agents treatment				
No	295/88	116/91	2.63(1.83,3.78)	2.18(0.98,4.81)
Yes	136/30	25/12	2.18(0.98,4.81)	2.46(0.93,6.54)
GCs treatment				
No	3/0	18/19	—	—
Yes	428/118	123/84	2.48(1.76,3.49)	2.74(1.81,4.14)
MMF treatment				
No	258/83	94/59	1.95(1.30,2.94)	2.40(1.25,4.61)
Yes	173/35	47/44	4.63(2.67,8.01)	5.34(2.57,11.1)
HCQ treatment				
No	50/11	38/27	3.23(1.43,7.32)	2.41(0.59,9.84)
Yes	381/107	103/76	2.63(1.82,3.79)	2.93(1.89,4.53)
CYC treatment				
No	101/29	77/59	2.67(1.56,4.55)	3.33(1.44,7.70)
Yes	330/89	64/44	2.55(1.63,4.00)	4.10(2.32,7.26)
Other immunosuppressants				
No	101/28	55/51	3.34(1.90,5.89)	4.85(2.12,11.09)
Yes	330/90	86/52	2.22(1.46,3.36)	3.28(1.89,5.70)

The confounding factors in the multivariate regression analysis were sex (female = 1, male = 0), age (continuous), SLE disease period (continuous), comorbidities (yes = 1, no = 0), SLEDAI score (continuous), abnormal urinary protein (yes = 1, no = 0), abnormal serum creatinine (yes = 1, no = 0), renal-protective agents (yes = 1, no = 0), MMF treatment (yes = 1, no = 0), CYC treatment (yes = 1, no = 0), HCQ treatment (yes = 1, no = 0), GCs dose (continuous) and other immunosuppressants (yes = 1, no = 0). The above adjusted variables would be excluded when they were the stratified variable

### Combined effects of TAC and other drugs on CR

Table 4 shows the results of TAC with CYC or MMF in SLE patients. The results show that the combination of TAC and CYC or MMF had better efficacy than CYC alone in SLE patients, where the adjusted ORs (95% CIs) were 2.15 (1.15, 4.02) and 2.43 (1.20, 4.92), respectively. Similarly, TAC combined with CYC or MMF had better efficacy than MMF alone, where the adjusted ORs (95% CI) were 3.14 (1.49, 6.64) and 3.54 (1.66, 7.58), respectively. A synergistic interaction was found between TAC and CYC or MMF with adjusted *P* values of 0.043 and 0.025, respectively.

**Table 4:** Effect of TAC with MMF or CYC on the complete response in SLE patients

TAC/CYC/MMF	Non-CR, *n* (%)	CR, *n* (%)	OR (95% CI)	Adjusted OR (95% CI)
**CYC as reference**				
CYC	258(75.7)	83(24.3)	1.00	1.00
TAC	51(58.6)	36(41.4)	1.72(1.04,2.85)	1.97(1.10,3.53)
TAC + CYC	43(65.2)	23(34.8)	2.02(1.16,3.51)	2.15(1.15,4.02)
TAC + MMF	26(53.1)	23(46.9)	2.75(1.49,5.08)	2.43(1.20,4.92)
TAC + MMF + CYC	21(50.0)	21(50.0)	3.11(1.62,5.98)	2.69(1.33,5.45)
*P*_TAC*CYC_			0.038	0.043
**MMF as reference**				
MMF	101(77.7)	29(22.3)	1.00	1.00
TAC	51(58.6)	36(41.4)	2.46(1.36,4.45)	2.89(1.50,5.58)
TAC + MMF	26(53.1)	23(46.9)	3.08(1.54,6.18)	3.54(1.66,7.58)
TAC + CYC	43(65.2)	23(34.8)	1.86(0.97,3.58)	3.14(1.49,6.64)
TAC + MMF + CYC	21(50.0)	21(50.0)	3.48(1.67,7.24)	3.94(1.74,8.95)
*P*_TAC*MMF_			0.013	0.025

The confounding factors in the multivariate regression analysis were sex (female = 1, male = 0), age (continuous), SLE disease period (continuous), comorbidities (yes = 1, no = 0), SLEDAI score (continuous), abnormal urinary protein (yes = 1, no = 0), abnormal serum creatinine (yes = 1, no = 0), renal-protective agents (yes = 1, no = 0), HCQ treatment (yes = 1, no = 0), GCs dose (continuous) and other immunosuppressants (yes = 1, no = 0). *P*_TAC*CYC_ is *P* for the synergy between TAC and CYC. *P*_TAC*MMF_ is *P* for the synergy between TAC and MMF

We analysed the efficacy of different doses of TAC in combination with MMF or CYC, and the results showed a dose-response relationship between TAC dosage and CR rates in combination therapy (Table 5). The efficacy was optimal when ≥ 4 mg/d TAC was combined with MMF or CYC for treatment, where the adjusted ORs (95% CIs) were 4.87 (1.17, 20.17) and 8.86 (2.31, 33.94), respectively.

**Table 5:** Effects of different doses of TAC with CYC or MMF on the complete response in SLE patients

TAC/CYC/MMF	Non-CR, *n* (%)	CR, *n* (%)	OR (95% CI)	Adjusted OR (95% CI)
**TAC/CYC**				
CYC	258(75.7)	83(24.3)	1.00	1.00
TAC ≤ 2 mg/d	28(58.3)	20(41.7)	2.22(1.19,4.15)	1.74(0.87,3.48)
TAC 3 mg/d	20(62.5)	12(37.5)	1.87(0.87,3.98)	1.54(0.67,3.57)
TAC 4 mg/d	3(42.9)	4(57.1)	4.14(0.91,18.9)	3.02(0.61,14.93)
TAC ≤ 2 mg/d + CYC	41(65.1)	22(34.9)	1.67(0.94,2.96)	1.62(0.80,3.32)
TAC 3 mg/d + CYC	19(55.9)	15(44.1)	2.45(1.19,5.05)	2.48(1.06,5.80)
TAC 4 mg/d + CYC	4(36.4)	7(63.6)	5.44(1.55,19.05)	4.87(1.17,20.17)
*P*_TAC dose*CYC_			0.001	0.009
**TAC/MMF**				
MMF	101(77.7)	29(22.3)	1.00	1.00
TAC ≤ 2 mg/d	28(58.3)	20(41.7)	2.49(1.23,5.04)	2.74(1.27,5.90)
TAC 3 mg/d	20(62.5)	12(37.5)	2.09(0.91,4.77)	2.41(0.99,5.85)
TAC 4 mg/d	3(42.9)	4(57.1)	4.64(0.98,21.94)	4.64(0.94,22.86)
TAC ≤ 2 mg/d + MMF	32(59.3)	22(40.7)	2.39(1.21,4.74)	2.49(1.10,5.64)
TAC 3 mg/d + MMF	11(47.8)	12(52.2)	3.8(1.52,9.5)	4.58(1.47,14.27)
TAC 4 mg/d + MMF	4(28.6)	10(71.4)	8.71(2.54,29.81)	8.86(2.31,33.94)
*P*_TAC dose*MMF_			0.012	0.011

The confounding factors in the multivariate regression analysis were sex (female = 1, male = 0), age (continuous), SLE disease period (continuous), comorbidities (yes = 1, no = 0), SLEDAI score (continuous), abnormal urinary protein (yes = 1, no = 0), abnormal serum creatinine (yes = 1, no = 0), renal-protective agents (yes = 1, no = 0), HCQ treatment (yes = 1, no = 0), GCs dose (continuous) and other immunosuppressants (yes = 1, no = 0). The MMF treatment (yes = 1, no = 0) was further adjusted in the TAC/CYC group, and the CYC treatment (yes = 1, no = 0) was adjusted in the TAC/MMF group. *P*_TAC dose*CYC_ is *P* for the synergy between different doses of TAC and CYC. *P*_TAC dose*MMF_ is *P* for the synergy between different doses of TAC and MMF

## Discussion

In this large retrospective cohort study, we confirmed that TAC was effective in achieving CR in SLE patients, where a higher rate was observed among those who received TAC more than 4 mg/d or more than 180 days. Furthermore, a synergistic interaction was first observed for the combination of TAC with MMF or CYC, where the CR rate was higher than those of each drug alone.

Previous studies have shown that TAC can reduce urinary protein and renal involvement, and its therapeutic effect is more significant than that of traditional immunosuppressants such as CYC and MMF [20, 21]. TAC may be effective at increasing complete response rates in SLE patients and has a dose-response relationship. However, few studies have investigated the dose-response effects of TAC. A five-year follow-up study of LN patients treated with TAC showed that the mean urinary protein/creatinine ratio annually decreased, and a meta-analysis showed that the efficacy of TAC increased over time [22, 23]. Although a dose-response relationship between TAC dose and CR was suggested, the TAC blood concentration was within the safe range with a median (IQR) TAC concentration of 3.00 (2.43, 5.00) ng/ml among TAC users in this study. There are intricate metabolic pathways, which encompass intricate modulation by gene polymorphisms such as CYP3A5, ABCB1, and MDR1, and the activity of P-glycoprotein. Thus, therapeutic drug monitoring is important throughout the TAC administration to ensure optimal and safe treatment outcomes [24]. 

TAC is effective in different populations, which supports its general effectiveness in improving renal function. This study reported significant therapeutic effects of TAC in patients with hypertension, diabetes, hyperlipidaemia, tumours, and secondary Sjogren’s syndrome. Furthermore, our findings revealed a substantial enhancement in the renal response among SLE patients who experienced moderate to severe increases in urinary protein levels when they were treated with TAC. Notably, prior investigations have not delved into the full spectrum of the therapeutic efficacy of TAC in SLE patients with diverse urinary protein manifestations. The significant effect of TAC may be due to TAC protecting the renal function and preventing protein loss by stabilizing the synaptic podocyte protein expression and podocyte cytoskeleton, preserving the podocyte numbers, reducing the podocyte apoptosis, inhibiting podocyte fusion, maintaining the podocyte integrity and protecting the glomerular filtration barrier [25–27]. 

In previous studies, the multi-target therapy of TAC with MMF or CYC was stronger than monotherapy [9, 17]. In this study, the combined effect was also better than that of monotherapy, and synergy was observed for the first time. In a meta-analysis of LN patients, the CR rate of TAC with MMF was 53.0%, which was significantly higher than the CR rate of 27.1% for TAC with CYC (*P*< 0.001) [9]. In a study of 368 patients, TAC with MMF had a significantly higher CR rate and fewer adverse reactions than CYC [15]. The optimal dosage of TAC combined with MMF in patients with SLE lacks evidence in the literature; however, our findings suggest that ≥ 4 mg/d TAC with MMF has superior efficacy. Consistent with our findings, a long-term cohort study also revealed that 4 mg/d TAC combined with MMF can be used to treat LN patients whose standard treatment is ineffective [28]. Thus, TAC combined with MMF treatment can effectively relieve the condition of SLE patients. This effect may result from MMF inhibiting the lymphocyte proliferation, antibody formation and IL-2 production when combined with TAC. In vivo studies have also shown that the combination of MMF and TAC significantly reduces the expression of serum TGF-β1 and cystatin C [29]. 

Although the CR rate of TAC with CYC was not as high as that of TAC with MMF in our study, TAC combined with CYC can remain an important option for multi-target drug therapy in clinical practice. For patients with refractory LN, multi-targeted therapy with TAC and CYC may be a potentially valuable approach. Previous studies have reported the successful treatment of LN patients who did not respond to IV-CYC via the addition of TAC [30]. Sakai’s study showed that the combination of TAC (3.0 mg/day) and CYC resulted in a significantly higher CR rate after 6 months than CYC alone [31]. Compared with previous studies, our study provided a clearer separation of TAC doses and confirmed the synergistic interaction among different doses of TAC and CYC. As an alkylating agent that inhibits cell proliferation, CYC primarily treats SLE patients by inhibiting the proliferation of T and B lymphocytes in patients and suppressing the lymphoblast response to antigen stimulation [32, 33]. The combination therapy of TAC and CYC may have a synergistic effect because they can inhibit both T and B lymphocytes. Although the treatment regimen of TAC with CYC has not been widely recognized and there is insufficient research on the pharmacological effects and treatment outcomes of these two drugs, our study has discovered for the first time a synergistic interaction between TAC and CYC.

Potential confounders, including age, weight, HCQ and renal-protective agents, significantly varied according to TAC status and were considered in the multivariate analysis to control for their influence. Although HCQ is widely used for SLE patients, not all patients used it during the study period [34]. because for patients with a prolonged disease duration, HCQ is generally discontinued after 5 years of oral administration to avoid secondary fundus toxicity [35]. Furthermore, patients who cannot tolerate the adverse effects of HCQ, such as rash, hair loss, and skin darkening, are not required to continue using it. We categorized certain herbs as renal-protective agents and incorporated them into our analysis as potential confounders, since previous studies highlighted their beneficial role in renal protection [36–40]. Previous studies reported the renal effects of ACEI and ARB in LN patients, so ACEI and ARB were also considered but not associated with renal response in this study, possibly due to the limited sample size (13.3% of all patients) [41]. Finally, Voclosporin, which is a novel calcineurin inhibitor, received approval from the FDA on January 22, 2021, and has demonstrated significant efficacy in patients with LN [42, 43]. Nevertheless, it is expensive and not available everywhere, so TAC remains an important conventional treatment option.

This study has several limitations. First, this was a retrospective observational study. Second, only 11.2% of patients underwent renal biopsy. Since renal biopsy is not an essential diagnostic criterion for SLE and is both costly and associated with risks, many patients avoid undergoing this procedure. Therefore, we defined our study participants as SLE patients with renal involvement, referring to the diagnostic criteria for LN proposed by the American College of Rheumatology. Third, although potential confounding factors were adjusted, it was impossible to collect all other factors, such as diet and family history, that might affect the renal function. Fourth, the assessment of renal response was based on hospital medical records, which may cause an underestimation of the renal response, as patients tend to seek medical attention due to disease flares or visit another medical institution that is not recorded by us. Fifth, the potential nephrotoxicity associated with TAC was not investigated in this study, and further research is necessary to assess the effects of long-term TAC use on the renal function and the synergy of TAC with other drugs. Despite these limitations, this cohort study has several strengths. This large retrospective study included over 790 Chinese SLE patients, which enabled multivariate regression analyses and stratified analyses to minimize potential confounding effects. For the first time, we reported the synergistic effects of TAC, MMF and CYC on the renal response and determined the dose-response relationship of TAC doses in this therapy.

## Conclusions

In this cohort study, TAC was effective in relieving the condition of SLE patients with a dose-response relationship in the dosage and duration of TAC use. In addition, TAC exhibited relieving efficacy in different subgroups of SLE patients, including SLEDAI score > 12, moderate or severe urinary protein and comorbidities. Compared to monotherapy, TAC with MMF or CYC was positively correlated with a higher CR rate, and a synergistic interaction was observed.

## Supplementary Information

Supplementary Material 1.

## Data Availability

The data presented in this study are available on request from the corresponding author following permission by the ethics committee of the hospital.
