# Supplementary material for: Effect of tacrolimus with mycophenolate mofetil or cyclophosphamide on the renal response in systemic lupus erythematosus patients
Source: BMC Rheumatol. 2024 Dec 18;8:68. doi: 10.1186/s41927-024-00439-x (PMC11654433; doi:10.1186/s41927-024-00439-x)
Supplement: Supplementary file 1 — Supplementary Material 1. [file 41927_2024_439_MOESM1_ESM.docx]

## SUPPLEMENTARY MATERIAL

**Supplementary Table 1.** Baseline clinical features of SLE patients after propensity score matching

| **Variable** | **TAC non-users** | **TAC users** | ***P*** |
| --- | --- | --- | --- |
| Total | n = 198 | n = 198 | — |
| Gender (Female), n (%) | 169(85.4) | 183(92.4) | **0.037** |
| Age, M (IQR), years | 31.0(25.0,41.0) | 29.0(24.0,37.0) | 0.769 |
| Weight, M (IQR), kg | 55.0(50.0,62.3) | 57.6(55.0,65.3) | **0.001** |
| SLE disease period, M (IQR), years | 0.0(0.0,2.0) | 1.0(0.0,3.0) | **0.036** |
| Renal biopsy, n (%) | 28(14.1) | 30(15.2) | 0.887 |
| SLEDAI score, M (IQR) | 16.0(10.0,22.0) | 15.0(11.0,20.0) | 0.588 |
| Follow-up duration, M (IQR), days | 134(94.0,255.0) | 119.0(91.0,214.0) | 0.098 |
| Comorbidities, n (%) |  |  |  |
| All | 177(89.4) | 174(87.9) | 0.752 |
| Hyperlipemia | 88(44.4) | 106(46.5) | 0.087 |
| Hypertension | 127(64.1) | 127(64.1) | 1.000 |
| Diabetes | 16(8.1) | 14(7.1) | 0.850 |
| Tumor | 10(5.1) | 9(4.5) | 1.000 |
| Secondary Sjogren’s syndrome | 7(3.5) | 2(1.0) | 0.175 |
| Indicators of renal involvement, n (%) | |  |  |
| Urinary protein |  |  |  |
| Mild | 28(14.1) | 27(13.6) | 0.969 |
| Moderate | 48(24.2) | 50(25.3) |  |
| Severe | 122(61.6) | 121(61.1) |  |
| Pathological tube pattern | 53(26.8) | 49(24.7) | 0.730 |
| Abnormal serum creatinine | 125(63.1) | 121(61.1) | 0.756 |
| Abnormal eGFR | 17(22.1) | 37(22.6) | 1.000 |
| Anti-dsDNA positive, n (%) | 68(34.3) | 37(18.7) | **0.001** |
| Low complement, n (%) |  |  |  |
| C3 ≤ 0.8 g/L | 38(20.7) | 60(30.3) | **0.035** |
| C4 ≤ 0.2 g/L | 27(14.7) | 75(37.9) | **<0.001** |
| Treatments |  |  |  |
| TAC length, M (IQR), days | — | 119.0(91.0,214.0) | — |
| TAC dose, M (IQR), mg/d | — | 2.0(0.0,3.0) | — |
| GCs, n (%) | 197(99.5) | 167(84.3) | **<0.001** |
| GCs maintenance dose, M (IQR), mg/d | 30.0(20.0,45.0) | 30(11.9,50.0) | 0.340 |
| MMF, n (%) | 84(42.4) | 71(35.9) | 0.217 |
| MMF dose, M (IQR), g/d | 1.0(1.0,1.5) | 1.0(1.0,1.5) | 0.681 |
| CYC, n (%) | 145(73.2) | 81(40.9) | **<0.001** |
| CYC dose, M (IQR), g/m^2^ | 0.4(0.3,0.4) | 0.4(0.2,0.4) | 0.117 |
| Renal-protective agents, n (%) | 50(25.3) | 34(17.2) | 0.065 |
| HCQ, n (%) | 180(90.9) | 146(73.7) | **<0.001** |
| Other immunosuppressants, n (%) | 146(73.7) | 117(59.1) | **0.003** |
| Leflunomide | 45(22.7) | 55(27.8) |  |
| Methotrexate | 5(2.5) | 2(1.0) |  |
| Tripterygium wilfordii | 4(2.0) | 9(4.5) |  |
| Azathioprine | 2(1.0) | 8(4.0) |  |
| Metronidazole | 0(0.0) | 2(1.0) |  |
| Sunitinib | 6(3.0) | 6(3.0) |  |
| M (IQR): median (interquartile range), eGFR: estimated glomerular filtration rate, Anti-dsDNA: anti-double stranded DNA, GCs: glucocorticoids, HCQ: hydroxychloroquine, MMF: mycophenolate mofetil, CYC: cyclophosphamide. The propensity score model included recipient's age, sex, SLEDAI score, abnormal urinary protein, and abnormal creatinine rate. | | | |
